# Klebsiella‐induced acute infectious purpura fulminans in a Thai woman: Case report and review of literature

**DOI:** 10.1002/ski2.186

**Published:** 2022-11-06

**Authors:** Bhakinai Temnithikul, Suthat Rungrunanghiranya, Piyakan Limtanyakul, Teeranan Angkananard, Vesarat Wessagowit

**Affiliations:** ^1^ Department of Medicine HRH Princess Maha Chakri Sirindhorn Medical Center, Srinakharinwirot University Ongkharak Nakhon Nayok Thailand; ^2^ HRH Princess Chulabhorn College of Medical Science Bangkok Thailand; ^3^ Institute of Dermatology Ministry of Public Health Bangkok Thailand

## Abstract

Purpura fulminans (PF) is an uncommon syndrome of acute purplish skin eruption characterized by coagulation of the microvasculature, which leads to purplish lesions and skin necrosis. There are three subtypes; idiopathic PF, neonatal PF and, the most common subtype, acute infectious PF (AIPF). Acute infectious PF is related to the thrombotic subtype of disseminated intravascular coagulation (DIC) and usually is superimposed on sepsis. This can rapidly lead to multi‐organ failure from thrombotic occlusion of small and medium‐sized blood vessels. We report a case of Klebsiella‐induced AIPF in a 78‐year‐old Thai woman and also review other published cases.

## Graphical abstract

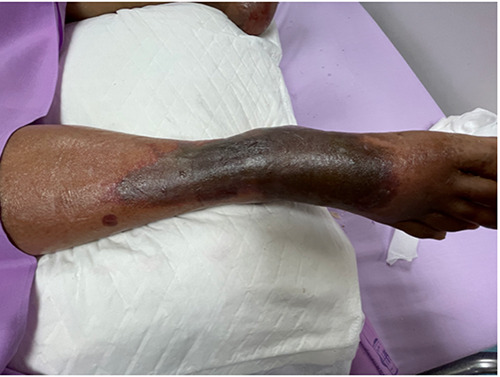

We described a rare unique case of an adult with Klebsiella‐induced AIPF. Only eight cases of PF have been reported in the setting of *Klebsiella* spp infection since 2002. Our case is the fourth case of *Klebsiella* pneumoniae‐induced AIPF, in which prompt antibiotic treatment and intensive care therapy are critical for the patient's survival.

## 1 CASE REPORT

A 78‐year‐old Thai woman was admitted to our department due to pneumonia with sepsis. She was brought to the intensive care unit due to shock and multi‐organ failure, requiring the use of mechanical ventilation and vasopressors. She was prescribed cefoperazone with sulbactam (sulperazone) and colistin.

**FIGURE 1:**
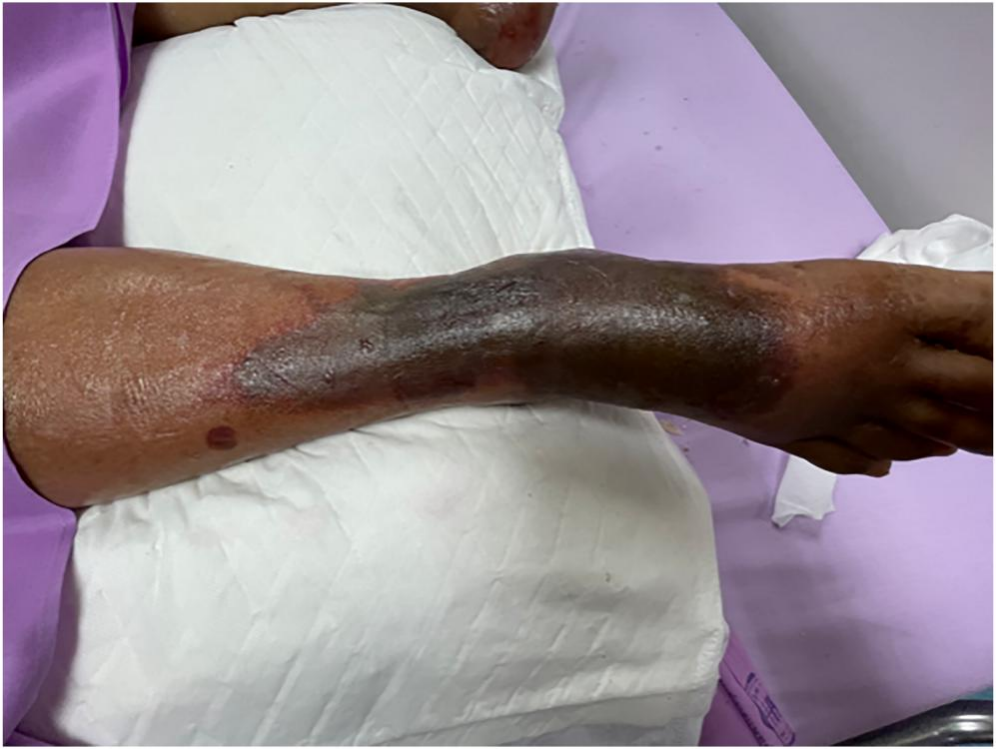
Multiple vesiculobullous on top of peripheral erythematous patches in purpuric rash on the left lower extremity

**FIGURE 2:**
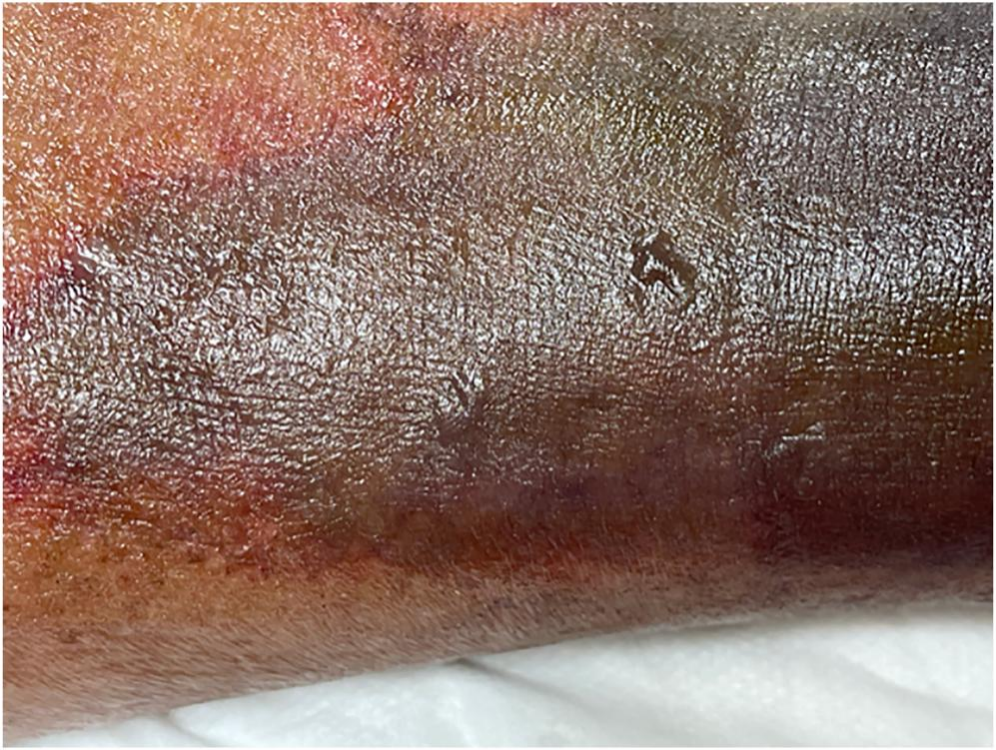
Multiple vesiculobullous lesions on top of peripheral erythematous patches in purpuric rash on the left lower extremity

On the third day of hospitalisation, physical examination revealed cold bilateral distal extremities and skin mottling in the left lower extremity. The skin mottling progressed, eventually resulting in a purpuric rash and bullae across the left lower leg [Figures 1 and 2]. Dermatology consultation was requested on the fifth day to confirm the clinical suspicion of acute infectious PF (AIPF). A biopsy was performed. Laboratory testing showed anaemia, thrombocytopaenia, a high prothrombin, time (PT)/partial, thromboplastin time (PTT) ratio [prolonged PT (24.80 s), prolonged PTT (48.70 s)] and international normalized ratio (2.27 s), all of which were suggestive of DIC.

At the time of presentation, two further differential diagnoses were considered: vasopressor‐induced necrosis and PF. Given her gangrenous left lower extremity, vasopressor‐induced necrosis was a possibility, but it was ruled out due to the haemorrhagic bullae development early on, fast progression to her lower extremities, and laboratory abnormalities indicative of DIC.

**FIGURE 3:**
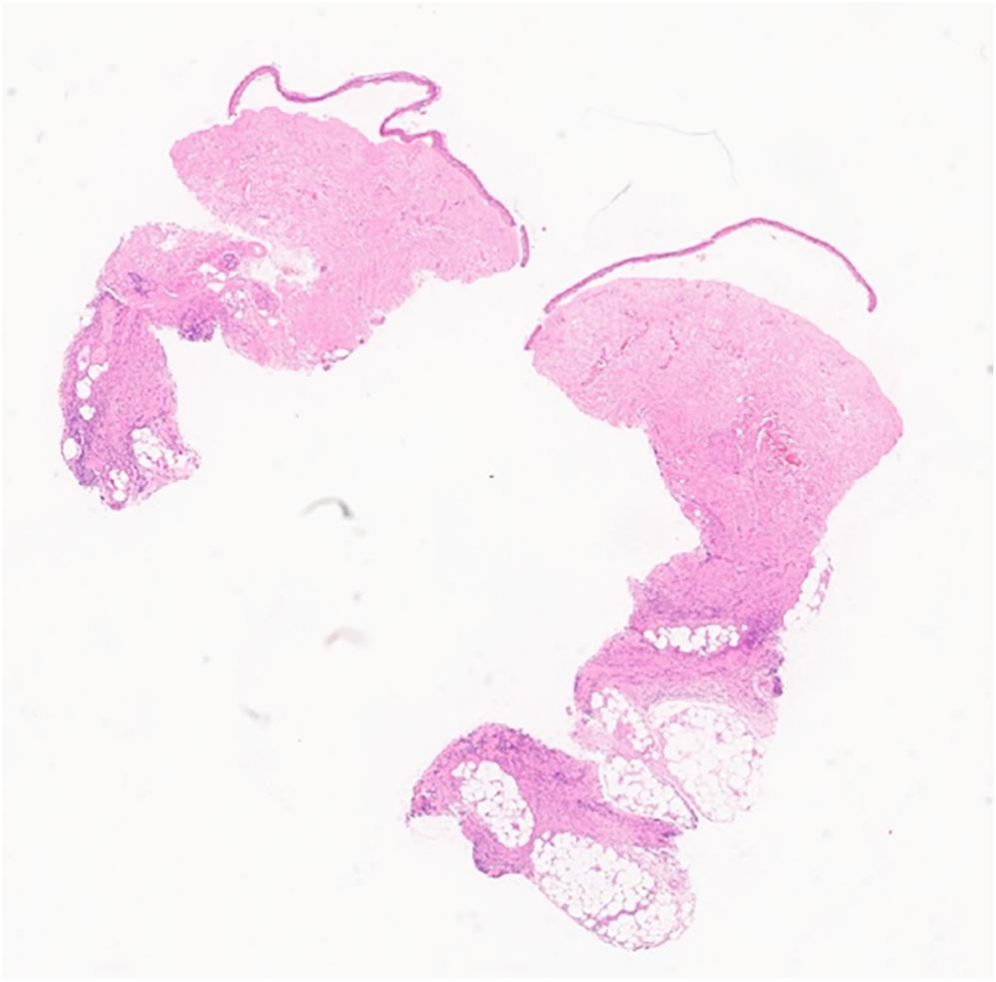
Subepidermal vesicle (H&E, x 4)

**FIGURE 4:**
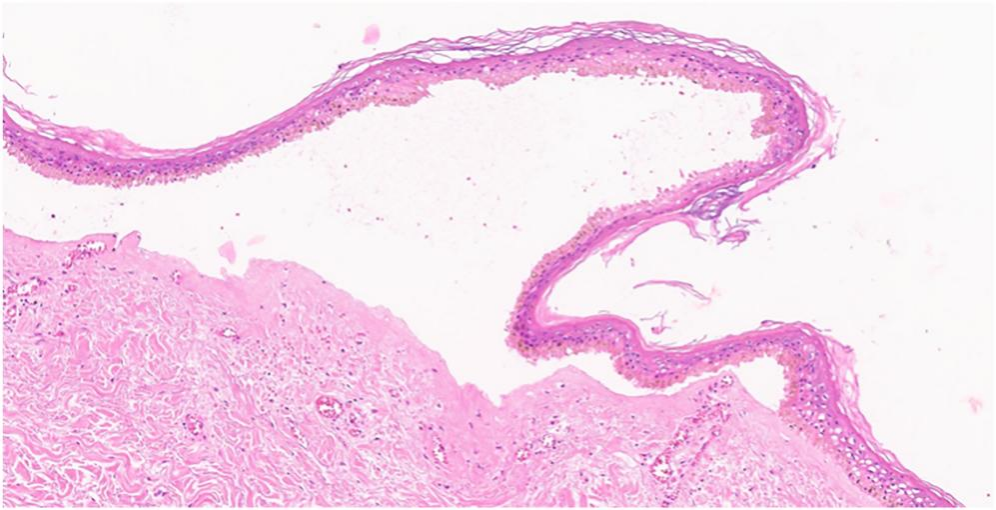
The overlying epidermis show confluent necrosis. (H&E, x 40)

**FIGURE 5:**
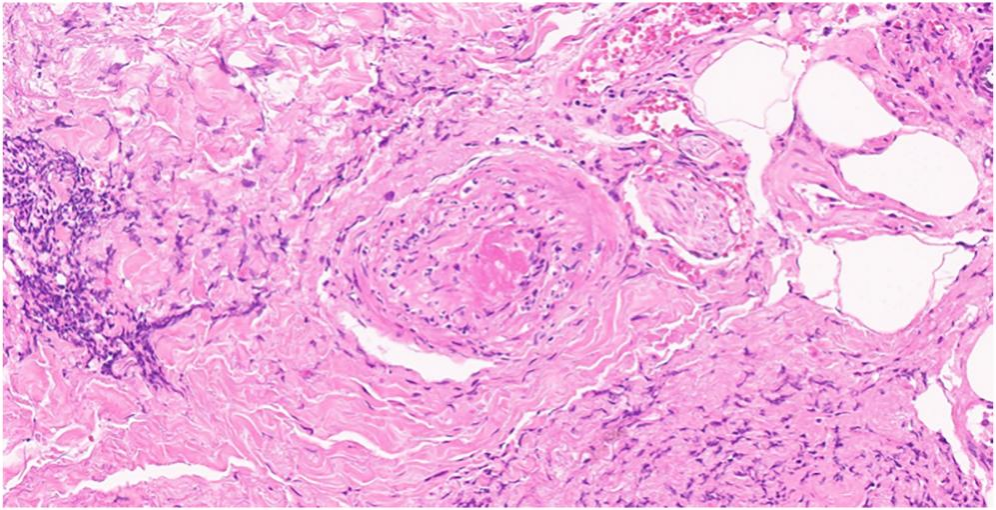
The dermis show fibrin thrombi in many small‐to medium‐sized blood vessels. (H&E, x 100)

**FIGURE 6:**
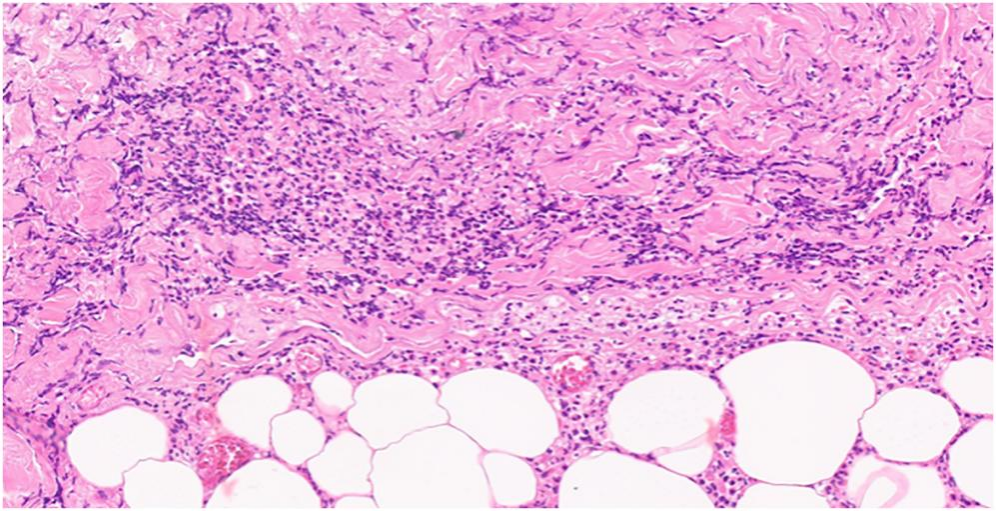
The dermis show a perivascular and interstitial mixed inflammatory cell infiltrate compose of lymphocytes and a few neutrophils in the dermis and subcutaneous tissue. (H&E, x 100)

Microscopic examination revealed confluent necrosis of the overlying epidermis, with a subepidermal vesicle that contained a few lymphocytes. The dermis showed a perivascular and interstitial mixed inflammatory cell infiltrate composed of lymphocytes and a few neutrophils in the dermis and subcutaneous tissue. Fibrin thrombi were seen in many small to medium‐sized blood vessels. The pathological diagnosis was epidermal necrosis with fibrin thrombi [Figure 3, 4, 5, 6].

Sputum cultures indicated positive growth for *Klebsiella pneumoniae* 2 days after pathology findings were reported, and blood cultures (VITEK 2 system) confirmed positive growth for *Klebsiella pneumoniae* sensitive to sulperazone and colistin. One week after empirical antibiotic treatment, sepsis and AIPF improved.

Based on a case of rapidly progressing retiform purpura, DIC, skin biopsy findings, and clinical improvement after antibiotic therapy, the most likely diagnosis in our patient was PF associated to *Klebsiella pneumoniae* sepsis.

## 2 DISCUSSION

Purpura fulminans is an uncommon syndrome of acute purplish skin eruption characterised by DIC, failure of the circulation and dermal vascular thrombosis, which leads to cutaneous haemorrhagic purplish lesions and skin necrosis in the trunk and extremities.^1^, ^2^, ^3^, ^4^, ^5^ PF is a life‐threatening disorder with a mortality rate up to 60%.^1^, ^6^ Idiopathic PF, neonatal PF and AIPF are the three subtypes.^2^, ^7^ The most prevalent subtype is AIPF which occurs in conjunction with sepsis and is linked to the thrombotic subtype of DIC.

This syndrome has significant death rate and is mostly seen in children, with only 4% of cases affected patients over the age of twelve.^8^ Fever, shock and systemic consumptive coagulopathy occur in affected patients, leading to multisystem organ failure,^9^ with Waterhouse‐Friderichsen syndrome (DIC, organ failure, and adrenal haemorrhage) as the leading cause of mortality.^10^ The most common infectious aetiologies are *Neisseria meningitidis*, *Haemophilus influenzae* and *Streptococcus pneumoniae*, although other species have also been implicated, as in Table 1.^1^, ^4^, ^11^, ^12^, ^13^, ^14^, ^15^ Amidst COVID 19 ongoing pandemic, incidences of AIPF from SARS‐CoV‐2 have been reported as a manifestation of COVID‐19.^14^, ^15^

**TABLE 1:** Aetiologies of Purpura fulminans (PF)^1^, ^4^, ^14^

	Organisms/Conditions
1. Infectious	
1.1 Bacterial:	*Capnocytophaga canimorsus* *Enterococcus faecalis* *Escherichia coli* *Haemophilus aegyptius* *Haemophilus influenza* *Klebsiella pneumonia* *Leptospira interrogans* *Neisseria meningitidis* *Pseudomonas aeroginosa* *Rickettsia rickettsii* *Staphylococcus aureus* *Streptococcus pyogenes* *Streptococcus pneumonia* *Vibrio parahaemolyticus*
1.2 Protozoal:	*Plasmodium falciparum*
1.3 Viral:	SARS‐CoV‐2 (COVID‐19) Rubeola Varicella Zoster
2. Post‐Infectious/Idiopathic	
2.1 Post‐infectious	Rubella Rubeola *Streptococcus* spp. Varicella
2.2 Autoimmune	Autoimmune connective tissue disease Autoimmune protein C deficiency Autoimmune protein S deficiency
2.3 Heritable PC pathway defects:	Severe protein C deficiency Severe protein S deficiency
3. Others	Coumarin‐induced skin necrosis

Acute infectious PF results from the crosstalk between the septic and coagulation cascades. Attenuation of thrombomodulin (TM), a cofactor of thrombin that activates protein C (PC) and plays a role in endogenous anticoagulation, is caused by the underlying infection.^3^ A lack of TM inhibits PC activation, allowing coagulation to proceed uncontrolled, which results in DIC.^16^ Two potential pathways for the decrease of TM function in response to infections have been postulated by in vitro investigations. While a few studies have shown that infections cause downregulation of TM gene activity, other recent researches suggest that the protein is cleaved from the endothelium surface via a post‐transcriptional process, leading to the loss of coagulation negative feedback loop.^16^, ^17^, ^18^

Patients with PF initially present with erythematous macules that quickly become indurated, non‐blanching petechiae, leading to ecchymoses and purpuric plaques on the trunk and extremities.^1^ Early lesions may resemble a livedo pattern with mottled skin.^1^ The most characteristic cutaneous finding of PF is diffused, non‐inflammatory retiform purpura from extensive microvascular occlusion.^19^ Bullae secondary to skin necrosis may be seen.^12^ Patients may present with ischaemic digits, extremities^3^, ^20^ or symmetrical peripheral gangrene which is characterized by symmetrical distal gangrene in absence of any large vessel occlusion and it is usually associated with critical illness.^14^

In cases of DIC, more severe phenotypes can be encountered such as symmetrical necrotic purpura, haemorrhagic bullae and/or widespread ecchymoses and epidermal necrosis.^5^ If left untreated, AIPF can progress to symmetrical peripheral gangrene, digital and/or limb amputations and end‐organ failure. This pattern rapidly develops into permanent full‐thickness necrosis of the skin within 24–48 h, as opposed to purpuric rashes found in other diseases, including immune thrombocytopenic purpura or thrombotic thrombocytopaenia purpura. Patients with AIPF may also be vulnerable to secondary infection.^1^, ^4^, ^21^

Prolonged plasma clotting times, thrombocytopaenia, decreased plasma fibrinogen concentration, increased plasma fibrin‐degradation products and microangiopathic haemolysis are all common laboratory findings in patients with PF.^1^, ^4^ This pattern of abnormalities, however, is not diagnostic of PF and may occur in DIC from other causes.^4^ In addition, Patients with PF often have high C‐reactive protein (CRP) levels while their erythrocyte sedimentation rate (ESR) is abnormally low.^1^ This “ESR‐CRP disassociation” is caused by relative or absolute hypofibrinogenaemia in the presence of DIC, resulting in a depressed ESR.^1^ PC and protein S levels are typically lower in PF patients.^22^, ^23^, ^24^ PC levels of less than 40% are especially indicative of PF.^23^ Table 2 shows the clinical and laboratory characteristics of PF.^1^

**TABLE 2:** Clinical and laboratory features of PF^1^

Physical examination, clinical and laboratory features of purpura fulminans	
1 Vital sign	Septic physiology +/− shock
2 Dermatological examination	Purpura or reticular rash/skin mottling (particularly over extremities)
3 Others	Meningeal signs Immunocompromised state
4 Laboratory	Evidence of DIC ‐ low platelets + fibrinogen ‐ elevated PT, PTT, d‐dimer ‐ Protein C activity ≤40% Elevated CRP and inappropriately low ESR

Abbreviations: CRP, C‐Reactive protein; DIC, disseminated intravascular coagulation; ESR, Erythrocyte Sedimentation rate; PT, prothrombin time; PTT, partial thromboplastin time.

Histopathologically, there is dermal vascular thrombosis with the presence of mixed microthrombi composed of fibrin, platelets, and leucocytes in the dermal blood vessels.^1^, ^3^, ^8^, ^9^ Microthrombi of tiny cutaneous arteries correspond histologically with the first clinical symptoms.^1^ Endothelial cell enlargement and capillary dilatation then follow, leading to cell separation and blood vessel rupture with bleeding into the dermal stroma.^3^ The characteristic presence of a perivascular neutrophilic infiltration differentiates AIPF from both postinfectious (idiopathic) and haemostasis‐induced (neonatal) PF.^12^

Despite improvement in our knowledge of the processes causing PF, clinical judgement is still required when diagnosing patients and recommending therapy. In the case of AIPF, antibiotic medication is used in conjunction with supportive care. There is currently no agreement on how to treat PF. Broad‐spectrum antibiotics, anti‐coagulants, PC concentrate, platelets and fresh frozen plasma (FFP) are some of the therapies that have been used.^1^, ^4^, ^5^, ^6^, ^7^ PF patients with DIC need immediate FFP (10–20 ml/kg every 8–12 h) to replenish pro‐coagulant and anti‐coagulant plasma proteins lost during DIC. AIFP cases with significant thrombocytopaenia (platelet count <50 × 10^9^/dl) and hypofibrinogenaemia (fibrinogen concentration <1 g/dl) may need additional platelet concentrates (10–15 ml/kg) or cryoprecipitate transfusions (5 ml/kg), especially if pathological bleeding is also present. At present, therapies focus on therapeutic anticoagulation and rapid replenishment of circulating antithrombotic proteins, while future treatments may leverage the cytoprotective benefits of the PC pathway in addition to the current standard of care.^16^

Because of rapid and severe disease progression, it is imperative for clinicians to provide correct diagnosis and treatment of PF. Warfarin‐induced skin necrosis, cryoglobulinaemic vasculitis, anti‐phospholipid syndrome, heparin‐induced thrombocytopaenia, meningococcal infection and Henoch‐Schönlein purpura are among the differential diagnoses.^1^

Infectious PF is an uncommon but deadly condition characterised by a thrombotic type of DIC, haemodynamic collapse, and haemorrhagic cutaneous necrosis. A delay in diagnosis can have serious clinical repercussions for the patient, including amputation of limbs and death. Despite advances in our knowledge of the processes behind PF, clinical judgement is still required when diagnosing patients and in recommending therapy.

In conclusion, we described a rare unique case of an adult with Klebsiella‐induced AIPF. This case highlights the significance of evaluating PF in adults with sepsis. Only eight cases of PF have been reported in the setting of *Klebsiella spp* infection since 2002, four being *K*. *pneumoniae* and two of *Klebsiella oxytoca* and one of *Klebsiella rhinoscleromatis* (Table 3). [Correction added on 24‐November‐2022, after first online publication: the number of cases was changed from six to eight in the preceding sentence.] Our case is the fourth case of *Klebsiella pneumoniae*‐induced AIPF, in which prompt antibiotic treatment and intensive care therapy are critical for the patient's survival. Only one out of four case reports was a neonate, whereas the rest happened in adult patients.

**TABLE 3:** Klebsiella‐induced acute infectious PF (AIPF)

Authors	Age, sex	Organisms	Management	Outcome
Nguyen V, et al (2020)^25^	56, female	*Klebsiella pneumoniae*	ICU heparin antibiotics	Improvement limbs successful preserved
Ghosh SK, et al (2020)^26^	66, female	*Klebsiella* (no specific)	Sudden‐onset shock syndrome	Survived
Ghosh SK, et al (2020)^26^	68, female	*Klebsiella* (no specific)	Pneumonia	Death
Tsubouchi N, et al. (2019)^27^	75, female	*Klebsiella oxytoca*	Intensive care	Death
Disse SC, et al. (2018)^6^	17 days, male	*Klebsiella oxytoca* sepsis from central venous catheter	Broad‐spectrum antibiotics, ventilation, diuretics, protein C substitution, burn protocol	Limbs successfully preserved with scarring
Singh P, et al. (2018)^7^	19, female	*K*. *pneumoniae*	IV fluids, broad‐spectrum antibiotics, platelets	Unknown
Umar LW, et al. (2017)^28^	2‐month, male	*K*. *pneumoniae*	Ceftriaxone, blood transfusions, FFP considered	Parents left against medical advice
Olowu WA, et al. (2002)^29^	3.5, female	*Klebsiella rhinoscleromatis*	Chloramphenicol and penicillin	Autoamputation of 4th 5th left toes Discharged
Our patient	78, female	*K*. *pneumoniae*	ICU Vasopressors, mechanical ventilation Sulfoperazone with colistin	Improvement and discharged

## CONFLICT OF INTEREST

The author declares that there is no conflict of interest that could be perceived as prejudicing the impartiality of the research reported.

## AUTHOR CONTRIBUTIONS

**Bhakinai Temnithikul**: Conceptualization (Lead); Data curation (Lead); Formal analysis (Lead); Funding acquisition (Equal); Investigation (Lead); Methodology (Lead); Project administration (Lead); Resources (Lead); Software (Lead); Supervision (Equal); Validation (Lead); Visualization (Lead); Writing – original draft (Lead); Writing – review & editing (Lead). **Suthat Rungrunanghiranya**: Supervision (Supporting); Visualization (Supporting). **Piyakan Limtanyakul**: Conceptualization (Supporting); Data curation (Equal); Formal analysis (Equal); Funding acquisition (Equal); Writing – original draft (Supporting); Writing – review & editing (Supporting). **Teeranan Angkananard**: Conceptualization (Supporting); Formal analysis (Supporting). **Vesarat Wessagowit**: Conceptualization (Supporting); Data curation (Supporting); Resources (Supporting); Supervision (Supporting); Validation (Supporting); Visualization (Supporting); Writing – original draft (Supporting); Writing – review & editing (Supporting).

## ETHICS STATEMENT

Not applicable.

## Data Availability

Data available on request due to privacy/ethical restriction.
